# Shenfu Injection Inhibits Cardiac Fibroblast Activation and Improves Myocardial Fibrosis by Down-regulating M1 Macrophage-Derived Exosomal miR-155-5p

**DOI:** 10.1007/s10557-026-07901-7

**Published:** 2026-06-18

**Authors:** Fei Wang, Siyuan Hu, Lin Li, Kun Lian, Junyu Zhang, Xinchun Li, Ge Fang, Zhixi Hu

**Affiliations:** 1 Department of Traditional Chinese Medicine, the First Affiliated Hospital of University of South China, Hengyang, Hunan China; 2 School of Sports Art, Hunan University of Chinese Medicine, Changsha, Hunan China; 3 Provincial Key Laboratory of Traditional Chinese Medicine Diagnostics, Hunan University of Chinese Medicine, Changsha, Hunan China; 4 Post-Graduate School, Hunan University of Chinese Medicine, Changsha, Hunan China; 5 Hunan University of Chinese Medicine Integrated Chinese Medicine Affiliated Hospital, Changsha, Hunan China; 6 School of Traditional Chinese Medicine, Hunan Traditional Chinese Medical College, Zhuzhou, Hunan China

**Keywords:** Cardiac fibrosis, M1 macrophage, Exosome, miR-155-5p, Shenfu Injection

## Abstract

**Purpose:**

Myocardial fibrosis is a key pathological feature of heart failure (HF) linked to aberrant macrophage polarization. Although the traditional Chinese medicine Shenfu injection (SFI) clinically improves HF and has been found to inhibit M1 macrophage polarization and fibrosis, its mechanisms remain unclear. This study aimed to elucidate how SFI regulates macrophage-cardiac fibroblasts (CFs) communication — specifically via exosomal miR-155-5p from M1 macrophages.

**Methods:**

An isoproterenol (ISO)-induced mouse model of HF was established to assess the effects of SFI on cardiac function, inflammatory cytokines, myocardial fibrosis, and the expression and localisation of miR-155-5p. *In vitro*, an M1 macrophage model was generated using lipopolysaccharide (LPS)-stimulated RAW264.7 cells, which were then treated with SFI or an miR-155-5p inhibitor. Exosomes from these cells were isolated and co-cultured with CFs to evaluate the miR-155-5p/SOCS1 pathway and fibrosis markers.

**Results:**

miR-155-5p expression was markedly upregulated and localized to infiltrating cardiac macrophages in mice. SFI treatment reduced levels of TNF-α, IL-6, and miR-155-5p, along with improved fibrosis and cardiac function. *In vitro*, SFI significantly decreased miR-155-5p levels in exosomes derived from M1 macrophages. Co-culture with exosomes from macrophages that were either SFI-treated or miR-155-5p-inhibited suppressed CFs activation. This was demonstrated by reduced expression of fibrosis markers (α-SMA, Col I, Col III), as well as decreased miR-155-5p levels and restored SOCS1 expression.

**Conclusion:**

This study demonstrates that SFI alleviates myocardial fibrosis by suppressing macrophage-derived exosomal miR-155-5p and restoring SOCS1 in CFs, thereby providing a mechanistic basis for its established clinical efficacy in HF. Our findings highlight the potential of targeting the macrophage-fibroblast miR-155-5p/SOCS1 axis as a therapeutic strategy.

**Graphical Abstract:**

**Figure:**
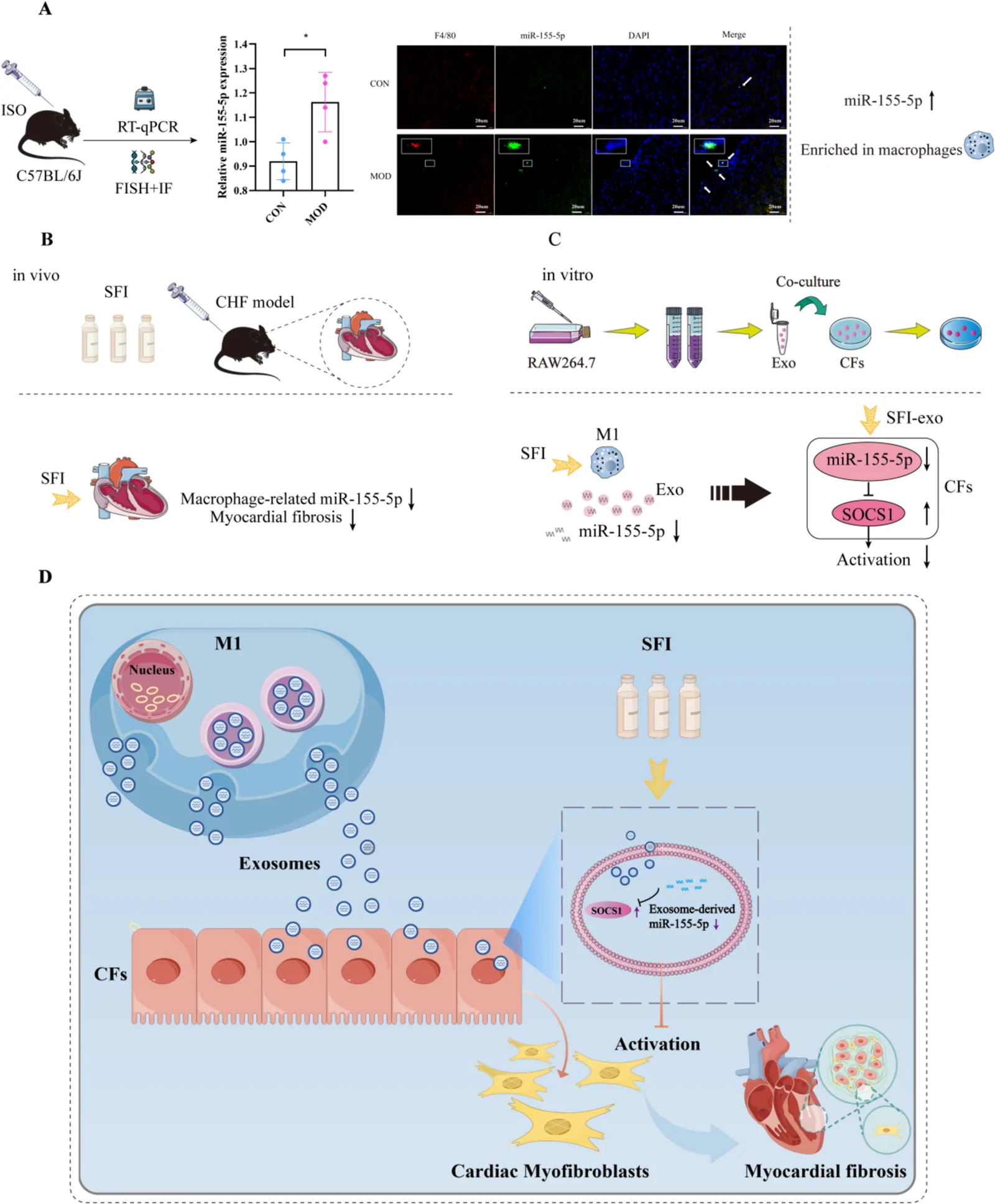
**A**: Upregulation of miR-155-5p in myocardial macrophages upon heart failure; **B**: SFI improves cardiac function and downregulates myocardial miR-155-5p *in vivo*; **C**: SFI inhibits CFs activation by repressing macrophage exosomal miR-155-5p. **D**: Schematic of the proposed molecular mechanism

**Supplementary Information:**

The online version contains supplementary material available at https://doi.org/10.1007/s10557-026-07901-7.

## Introduction

Heart failure (HF) is a clinical syndrome characterized by cardiac structural or functional impairments that hinder either ventricular systolic or diastolic function [1]. The aetiology of HF is multifaceted, encompassing hypertension, cardiomyopathy, ischaemic, and valvular heart diseases [2]. Conventional management of HF involves diuretics, β-blockers, and angiotensin-converting enzyme inhibitors. However, despite advances in the treatment and management of HF, it continues to be a leading cause of death and a major reason for hospital admissions, contributing significantly to the burden on healthcare and financial strain [3]. Consequently, it is crucial to further investigate the underlying mechanisms of HF, explore complementary and alternative therapies, and identify novel therapeutic targets.

Characterized by CFs differentiation and extracellular matrix deposition, myocardial fibrosis reduces cardiac elasticity and compliance, impairing the heart’s normal systolic and diastolic functions. This process constitutes a pivotal pathological aspect of HF [4, 5]. Myocardial fibrosis is known to be closely associated with immune inflammation, of which macrophages are the primary regulators. During myocardial injury, tissue-resident macrophages undergo phenotypic and functional shifts that can perpetuate tissue damage, leading to excessive fibrosis and HF [6]. Exosomes are microscopic vesicles released by cells and contain proteins and nucleic acids, which facilitate intercellular communication. Recent studies have demonstrated that tissue-resident macrophages transition to the M1 phenotype following myocardial injury [7]. They release exosomes to facilitate communication between macrophages and fibroblasts, thereby promoting the transformation of quiescent fibroblasts into myofibroblasts [8].

MicroRNAs (miRNAs) regulate gene expression post-transcriptionally and are pivotal active molecules transmitted between cells via exosomes, consequently exerting regulatory roles. miR-155-5p, as a multifunctional miRNA, was initially implicated in biological processes, such as inflammatory responses, immune responses, and developmental differentiation of immune cells [9–11]. Subsequently, substantial evidence has demonstrated the crucial involvement of miR-155-5p in the development of fibrosis [12–14]. Moreover, research showed that miR-155-5p is primarily secreted by activated macrophages and is transported to CFs via exosomes, where it is expressed and effectively influences functional outcomes [8]. The overexpression of miR-155-5p facilitates CFs proliferation, consequently increasing the expression of Col I and α-SMA markers [15]. Additionally, the suppressor of cytokine signalling 1 (SOCS1) is a downstream target of miR-155-5p, which interacts via base complementary pairing, degrading SOCS1 mRNA and downregulating SOCS1 protein levels [16]. Research has found that the miR-155-5p/SOCS1 inflammatory pathway is closely associated with fibrosis and promotes its occurrence in different organs, involving the oral cavity, kidney, and liver [17–19]. However, whether miR-155-5p directly targets SOCS1 in CFs to promote myocardial fibrosis has not been fully established.

Traditional Chinese Medicine has displayed specific benefits in managing HF [20]. Shenfu Injection (SFI), a modern preparation derived from the classical formula “Shenfu Decoction” (Song Dynasty, Ji Sheng Fang), is prepared from the extracts of *Red Ginseng* and *Radix Aconiti Lateralis Preparata*. SFI is recommended as an adjunctive therapy in a regional expert consensus on the integrated traditional Chinese and Western medicine management of chronic heart failure [21] and systematic reviews have summarized the clinical evidence regarding its efficacy and safety [22–24]. As myocardial fibrosis drives HF progression, investigating SFI’s anti-fibrotic mechanisms directly elucidates its therapeutic action. Modern pharmacological studies have demonstrated that SFI possesses multiple biological activities, including anti-inflammatory, antioxidant, and immunomodulatory effects, as well as enhancing myocardial contractility [25–28]. Our recent research has demonstrated that SFI reduces inflammatory reactions, alleviates myocardial fibrosis, and enhances heart functions in HF rats [29]. In a separate study using a mouse model, we have further found that SFI inhibits macrophage M1 polarization [30]. However, the exact mechanism by which SFI ameliorates myocardial fibrosis through immunomodulation, and how this process involves macrophages, remains unknown. We hypothesise that SFI attenuates the activation of CFs and inhibits myocardial fibrosis through downregulating the expression of M1 macrophage-derived exosomal miR-155-5p, which targets the SOCS1 signalling pathway.

This study intends to explore comprehensive mechanisms of M1 macrophage-derived exosomes in myocardial fibrosis as well as the ameliorative impacts of SFI. We elucidated the correlation between the anti-fibrotic properties of SFI and miR-155-5p expressed by exosomes derived from M1 macrophage utilising inflammation as a key link. We determined whether these effects are mediated through the miR-155-5p/SOCS1 route. Our findings revealed that SFI downregulated the miR-155-5p levels in M1 macrophage exosomes and targeted the SOCS1 pathway, thereby hindering the proliferation of CFs. Furthermore, this work provides a robust experimental basis for employing SFI for the management of HF in healthcare sectors.

## Materials and Methods

### Animal Experiments

The male C57BL/6J mice were supplied by Hunan Slack Jingda Experimental Animal Co., Ltd. (Hunan, China). They were 7–8 weeks old and weighed 20–22 g. These mice were housed in a 23 ± 1 °C environment with a 12 h dark or light cycle. They were fed a standard chow and water ad libitum. Approval was granted by the Research Ethics Committee of the Hunan University of Chinese Medicine (LLBH-202208240001).

### Quality Control of the SFI

SFI (batch number: 220505AK01) was purchased from China Resources Sanjiu Pharmaceutical Company Limited (Ya’an, Sichuan, China). It is formulated from *Aconitum carmichaelii* and *Panax ginseng* C. A. Mey (Table S1). According to the manufacturer, fingerprint identification technology is employed during production to ensure quality, and the entire process complies with the National Medical Products Administration of China (approval number: WS3-B-3427-98-2013) (Table S2). The preparation workflow is illustrated in Supplementary Fig. S1. To verify the quality of the specific batch used in this study, we performed Ultra-High Performance Liquid Chromatography coupled with Quadrupole-Orbitrap Mass Spectrometry (UHPLC-QE-MS) (Supplementary Fig. S2). Briefly, separation was achieved on a Waters UPLC BEH C18 column using a Vanquish UHPLC system (Thermo Fisher Scientific) [31]. Samples (5 µL injection volume) were eluted at a flow rate of 0.5 mL/min using a mobile phase consisting of 0.1% (v/v) aqueous formic acid (A) and acetonitrile (B) under elution gradient. MS and MS/MS data were acquired using a Q Exactive Focus mass spectrometer operated in information-dependent acquisition mode, covering a mass range of m/z 100–1500. MS/MS analysis was performed on the three most intense ions dynamically selected per acquisition cycle.

### HF Induction and Experimental Design

Sixty mice were randomly assigned to the control (*n* = 20) and model groups (*n* = 40). Based on a previously published protocol [32], 7.5 mg/kg of isoproterenol (ISO, HY-B0468/CS-2582, MedChemExpress, Monmouth Junction, NJ, USA) dissolved in physiological saline was injected intraperitoneally into the model group for 28 consecutive days to induce HF. An equal volume of physiological saline was injected into the mice in the control group. After the induction period, 10 mice from each group were randomly selected for model validation by echocardiography, serum NT-proBNP measurement, and histopathological examination (Supplementary Fig. S3). The remaining 30 model mice were further randomized into three subgroups (*n* = 10 each): MOD, SFI (Shenfu injection, 9 mL/kg, intraperitoneally), and CAP (captopril, H43020880, Hunan Xiangya Pharmaceutical Co., Ltd., Changsha, Hunan, China, 16.5 mg/kg, intragastrically) groups. CON and MOD mice received distilled water intragastrically and saline intraperitoneally; SFI mice received distilled water intragastrically and SFI intraperitoneally; CAP mice received captopril intragastrically and saline intraperitoneally. All treatments were administered once daily for 15 days. CAP was used as the positive control due to its well-established anti-fibrotic efficacy in ISO-induced heart failure models [33, 34]. No mice died during ISO induction, whereas one mouse in the MOD group died during the 15-day treatment period. At the end of treatment, blood and heart tissues were collected for biochemical, histopathological, and molecular analyses.

### Echocardiography and Cardiac Parameters

The mice were initially anaesthetised through induction with 4% isoflurane (22081302, Shenzhen RWD Life Science Co., Ltd., Shenzhen, Guangdong, China) at a flow rate of 4 mL/min. Then, they were secured in a supine position on a surgical board, with a heated padset at approximately 37 °C placed beneath them to maintain body temperature. The thoracic hair was shaved to expose the left thoracic and sternum, and anaesthesia was maintained with 1%-2% isoflurane. Throughout the imaging procedure, the isoflurane concentration was carefully titrated to maintain the heart rate within the physiologically acceptable range of 400–500 beats per minute. Transthoracic echocardiography was performed using a 6LAB portable ultrasonic device specifically engineered for small animal studies. Cardiac systolic function was evaluated through the measurement of the left ventricular ejection fraction (LVEF) and the left ventricular fractional shortening (LVFS). Moreover, the left ventricular end-diastolic diameter (LVEDD) and the left ventricular end-systolic diameter (LVESD) were assessed by calculating the mean values derived from signals obtained over three consecutive cardiac cycles.

### Determination of Cardiac Mass Index

Following the administration of the drugs, the mass of the mice was recorded. After euthanasia, hearts were excised, meticulously rinsed with PBS buffer to eliminate residual blood, and then carefully blotted dry with a filter paper before weighing. The heart mass index was quantified by taking a proportion of the mass of the heart to body weight.

### Serum NT-proBNP, IL-6, and TNF-α Detection

Following blood collection, the serum was isolated via centrifugation at 3000 rpm for 10 min at an ambient temperature. Following the manufacturer’s protocol, the levels of serum NT-proBNP (20230829, Jiangsu Jingmei Biotechnology Co., Ltd., Yancheng, Jiangsu, China), IL-6 (AF2163-A, Jiangsu Jingmei Biotechnology Co., Ltd.), and TNF-α (AF2132-A, Jiangsu Jingmei Biotechnology Co., Ltd.) were measured with commercially available diagnostic kits in mice.

### Histological Studies

Cardiac tissues were fixed in 4% paraformaldehyde, embedded in paraffin, and sectioned at 5 μm thickness. Three non-consecutive sections were prepared per animal, and were stained with hematoxylin and eosin (G1005, Wuhan Servicebio Technology Co., Ltd., Wuhan, Hubei, China) or Masson’s trichrome (abs9347, Absin Bioscience Co., Ltd., Shanghai, China). Images were acquired using a light microscope (Motic BA210T, Motic, Xiamen, China) coupled with a digital camera at 200× magnification. Three random fields per section were analysed by an investigator blinded to the experimental groups. The fibrotic area (blue staining in Masson’s trichrome) was calculated as the percentage of total myocardial area using ImageJ software (version 1.53c, National Institutes of Health, Bethesda, MD, USA). Three animals per group were analysed (*n* = 3). Data are expressed as mean ± SD. One-way ANOVA followed by Tukey’s HSD post hoc test was used for multiple comparisons (GraphPad Prism 8). A value of *P* < 0.05 was considered statistically significant.

### RNA Isolation and Real-time qPCR

Real-time quantitative reverse transcription PCR (RT-qPCR) was employed to analyse differentially expressed genes. Total RNA was extracted using TRIzol Reagent (15596026, Invitrogen, Carlsbad, CA, USA). RNA concentration and purity were determined using a NanoDrop spectrophotometer (Thermo Fisher Scientific, Waltham, MA, USA). Subsequently, 200 ng of total RNA was reverse transcribed into complementary DNA (cDNA) using the SuperScript III Reverse Transcriptase kit (11752050, Invitrogen, Carlsbad, CA, USA) according to the manufacturer’s protocol. Real-time PCR was carried out on a StepOne Real-Time PCR System (Applied Biosystems, Foster City, CA, USA) using SYBR Select Master Mix (4472920, Applied Biosystems, Foster City, CA, USA). The primer sequences used were recorded as follows: miR-155-5p: forward 5’-TCGGCAGGTTAATGCTAATTGTGAT-3’ and reverse 5’-CTCAACTGGTGTCGTGGAGT-3’; U6: forward 5’-CTCGCTTCGGCAGCACATATACT-3’ and reverse 5’-ACGCTTCACGAATTTGCGTGTC-3’; SOCS1:forward 5’-CGGTCAGAGCCCTCCTCGTCC-3’ and reverse 5’-GTCCGCGTGATGCGCCGGTA-3’; β-actin: forward 5’-CTCCTGAGCGCAAGTACTCT-3’ and reverse 5’-TACTCCTGCTTGCTGATCCAC-3’.

### Western Blot Analysis

Total protein was extracted from tissues, cells, and exosomes using RIPA buffer supplemented with a protease inhibitor cocktail. Equal amounts of protein were separated by SDS-PAGE on 10–12% polyacrylamide gels and transferred onto PVDF membranes. The membranes were blocked with 5% nonfat dry milk in TBST (0.05% Tween-20) for 1 h at room temperature and then incubated overnight at 4 °C with primary antibodies. After washing, the membranes were incubated with the appropriate HRP-conjugated secondary antibodies for 1 h at room temperature: goat anti-rabbit IgG (C030212, 1:5000, Servicebio, Wuhan, Hubei, China) or goat anti-mouse IgG (C030205, 1:5000, Servicebio, Wuhan, Hubei, China). Signals were detected using an enhanced chemiluminescence detection kit (E412-01, Vazyme Biotech Co., Ltd., Nanjing, Jiangsu, China) and a chemiluminescence imaging system. β-actin (AF7018, Affinity Biosciences, Cincinnati, OH, USA) was used as the internal control throughout, with specific dilutions noted for each experiment below.

For *in vivo* experiments, the following primary antibodies from Affinity Biosciences (Cincinnati, OH, USA) were used: anti-α-SMA (AF1032, 1:500), anti-Collagen I (Col I, AF7001, 1:500), anti-Collagen III (Col III, AF5457, 1:1000), and anti-SOCS1 (AF5378, 1:500). β-actin was diluted at 1:3000.

For *in vitro* experiments, including the rescue experiment, the primary antibodies used were as follows: anti-α-SMA (AF1032, 1:1000) and anti-Collagen I (AF7001, 1:1000) from Affinity Biosciences; anti-Collagen III (A12860, 1:1000) and anti-SOCS1 (A21727, 1:1000) from Alpha Applied Bioscience (Wuhan, Hubei, China). β-actin was diluted at 1:1000.

For exosome characterization, the membranes were probed with anti-TSG101 (TSG101, mEXOWBTSG101-5, 1:200), anti-ALIX (ALIX, mEXOWBALIX-5, 1:200), and anti-CD63 (CD63, mEXOWBCD63-5, 1:500) from Rengen Biosciences Co., Ltd. (Liaoning, China).

### Dual-labeling Staining Using Fluorescence in Situ Hybridization (FISH) and Indirect Immunofluorescence (IF)

Paraffin-embedded tissue sections were dewaxed and hydrated for antigen retrieval using a citrate buffer solution. Afterward, they were treated with hydrogen peroxide (3%) to suppress endogenous peroxidase activity and blocked with goat serum (10%). The procedure continued with an overnight incubation of the primary antibody at 4 °C. Secondary antibody was incubated at 37 °C for 60 min using the TSA signal amplification technique. The sections were again treated with proteinase K digestion and citrate for antigen retrieval. Subsequently, probe pre-hybridisation was performed for 60 min at 37 °C, then an overnight hybridisation was performed at 42 °C. The sections were washed in SSC solutions, and counterstained with DAPI staining solution (PN0015, Shanghai Pinofei Biotechnology Co., Ltd.). They were mounted using an anti-fade mounting medium (0100-01, SouthernBiotech, Birmingham, AL, USA), and a Nikon inverted fluorescence microscope (ECLIPSE Ci, Nikon Corporation, Tokyo, Japan) was utilised for observations.

### Cell Culture and Co-culture Experiment

The mouse monocyte-macrophage cell line RAW264.7 (CM-0190, Procell Life Science & Technology Co., Ltd., Wuhan, Hubei, China) and CFs (CP-M074, Procell Life Science & Technology Co., Ltd.) were sourced from Wuhan Procell Life Science & Technology Co., Ltd. All cells were grown in a humidified incubator in a 5% CO_2_ environment at 37 °C.

M1 polarization model establishment. RAW264.7 cells were seeded into 6-well plates at a density of 5 × 10^5^ cells per well in 2 mL of specific medium. After 24 h of culture, RAW264.7 cells were treated with 100 ng/mL lipopolysaccharide (LPS, L8880-10 mg, Beijing Solarbio Science & Technology Co., Ltd., Beijing, China) for 24 h to induce M1 polarization [35].

Experimental grouping and treatment. For the main experiments, RAW264.7 cells were divided into five groups according to the following protocols: the control group (CON group, receiving no drug treatment), the lipopolysaccharide group (LPS group, stimulated with LPS for 24 h), the miR-155-5p inhibitor group (miR-155-5p inhibitor group, transfected with miR-155-5p inhibitor for 48 h, then stimulated with LPS for 24 h), the Shenfu injection group (SFI group, pretreated with 4 µL/mL SFI for 24 h, followed by LPS for an additional 24 h), and the miR-155-5p inhibitor negative control group (inhibitor NC group, transfected with the miR-155-5p inhibitor NC for 48 h, then stimulated with LPS for 24 h). Conditioned media were collected from each group, and exosomes were isolated accordingly. CFs were then co-cultured with exosomes derived from the respective groups: CON-exo, LPS-exo, SFI-exo, inhibitor NC-exo, and miR-155-5p inhibitor-exo.

### Rescue Experiment

CFs were seeded in 12-well plates at a density of 2 × 10⁵ cells per well and cultured at 37 °C in a 5% CO₂ atmosphere. Cells were divided into three groups: (1) CON group: co-transfected with oe-NC and mimic-NC; (2) miR-155-5p mimic group: co-transfected with oe-NC and mmu-miR-155-5p mimic (50 nM); (3) miR-155-5p mimic + oe-SOCS1 group: co-transfected with a SOCS1 overexpression plasmid (oe-SOCS1; pcDNA3.1(+)-m-SOCS1, lacking the 3′UTR, 657 bp, HindIII-EcoRI, GenBank: NM_001271603.1; G96749-1, GenCefe, Jiangsu, China) (4 µg/well) and mmu-miR-155-5p mimic (50 nM). All transfections were performed using Lipofectamine 3000 according to the manufacturer’s instructions. After 48 h, cells were harvested, and the protein levels of α-SMA, Col I, and Col III, SOCS1 were analyzed by Western blot. β-actin was used as an internal control.

### Exosome Isolation

RAW264.7 macrophages were cultured in exosome-free serum-free medium for 48 h. The cell culture supernatant was collected and centrifuged at 3000 × g for 10 min at 4 °C to remove cells and debris, yielding conditioned medium.

Exosomes were isolated from the cleared supernatant using a commercial exosome extraction kit (EXOCon40-10, Rengen Biosciences Co., Ltd., Liaoning, China) according to the manufacturer’s protocol. Briefly, the supernatant was mixed with binding buffer and binding resin, incubated at room temperature with gentle rotation, and then centrifuged. The resin-bound exosomes were transferred to a purification column, washed, and eluted with elution buffer. The eluate was further concentrated by ultracentrifugation. The final exosome pellet was resuspended in PBS and stored at − 80 °C until further analysis.

### Cell Counting Kit-8 (CCK-8) Assay

The CCK-8 assay was performed using a CCK-8 kit (E-CK-A362, Elabscience Biotechnology Co., Ltd., Wuhan, Hubei, China) following the manufacturer’s protocol. For screening the optimal concentration of SFI: RAW264.7 macrophages were seeded into 96-well plates at a density of 3 × 10³ cells per well in 100 µL of culture medium. After overnight attachment, the cells were pretreated with various concentrations of SFI (1, 2, 4, 8, 12, and 16 µL/mL) for 24 h, followed by stimulation with 100 ng/mL LPS for an additional 24 h. For screening the optimal exosome co-culture conditions: CFs were seeded at the same density and treated with macrophage-derived exosomes at final concentrations of 25, 50, and 100 µg/mL for 12, 24, or 48 h.

After the respective treatments, 10 µL of CCK-8 solution was added to each well (final volume 100 µL) and the plates were incubated at 37 °C in the dark for 60 min. Absorbance was measured at 450 nm using a microplate reader (Multiskan FC, Thermo Fisher Scientific, Waltham, MA, USA).

### Cell Transfection

RAW264.7 macrophages (2 × 10⁴ cells/well) and CFs (2 × 10⁵ cells/well) were seeded in 12-well plates and cultured overnight. After replacing the medium with serum-free Opti-MEM (31985070, Gibco, Thermo Fisher Scientific, Waltham, MA, USA), cells were transfected with 50 nM of the indicated oligonucleotides using Lipofectamine 3000 (L3000015, Invitrogen, Carlsbad, CA, USA) according to the manufacturer’s protocol. RAW264.7 cells were transfected with miR-155-5p inhibitor (mi230661, RiboBio Co., Ltd., Guangzhou, China) or inhibitor NC (mi230662, RiboBio); CFs were transfected with miR-155-5p mimic, miR-155-5p inhibitor, or their respective negative controls (mimic NC, inhibitor NC), all synthesized by GENCEFE (Jiangsu, China). Cells were harvested 48 h after transfection. Transfection efficiency was confirmed by qPCR (Supplementary Fig. S4), and validated cells were used for subsequent experiments.

The sequences were as follows: miR-155-5p mimics: sense 5′-UUAAUGCUAAUUGUGAUAGGGGU-3′ and antisense 5′-CCCUAUCACAAUUAGCAUUAAUU-3′; miR-155-5p inhibitor: sense 5′-mAmCmCmCmCmUmAmUmCmAmAmUmUmAmGmCmAmUmUmAmAmUmUmU-3′ (where ‘m’ indicates 2′-O-methyl modification). Negative control sequences were universal scrambled sequences: mimics NC: sense 5′-UUUGUACUACACAAAAGUACUG-3′ and antisense 5′-CAGUACUUUUGUGUAGUACAAA-3′; inhibitor NC: 5′-CAGUACUUUUGUGUAGUACAAA-3′.

### Exosome Identification, Labelling, and Uptake

Firstly, exosomes were observed with a transmission electron microscope. Specifically, 10 µL of exosomes were placed in a copper grid, followed by liquid removal for 3 min. Subsequently, 50 µL of uranyl oxalate dihydrate solution was introduced for 5 min. After drying in the air, the images were captured under the transmission electron microscope. To quantify the concentration and diameter of the exosomes, nanoparticle tracking analysis was applied. Lastly, the marker proteins of exosomes were identified using the western blotting method.

Uptake of exosomes by CFs: According to the manufacturer’s guidelines, exosomes were labeled with PKH26 (HY-D1451, MedChemExpress, Monmouth Junction, NJ, USA) fluorescence. Subsequently, the labeled exosomes (50 µg/mL) were incubated with CFs for 24 h. Thereafter, the cells were rinsed using PBS and stabilised using 4% paraformaldehyde. After adding DAPI solution for nuclear staining, the cells were rinsed with PBS, and an antifade mounting medium was applied. Observations and photographs were taken under a fluorescence microscope.

### Dual-luciferase Reporter Assay

The wild-type (WT) SOCS1 3′UTR containing the predicted miR-155-5p binding site and a mutant (Mut) version with a scrambled seed sequence were cloned into the psiCHECK2 vector (Hanbio Biotechnology (Shanghai) Co., Ltd.). 293T cells (1 × 10⁴ per well) were seeded in 96-well plates and cultured until 50%–70% confluence. Cells were co-transfected with 0.16 µg of the WT or Mut reporter plasmid and 5 pmol of mmu-miR-155-5p mimic or mimic NC using a transfection reagent (Hanbio Biotechnology (Shanghai) Co., Ltd.) according to the manufacturer’s protocol. Fresh medium was replaced at 6 h post-transfection. After 48 h, firefly and Renilla luciferase activities were measured using the Dual-Luciferase Reporter Assay Kit (Hanbio Biotechnology (Shanghai) Co., Ltd.). Relative luciferase activity was expressed as the ratio of firefly to Renilla luciferase activity. All experiments were performed in triplicate.

### Flow Cytometry

Cultured macrophages were prepared as a single-cell suspension, counted, and acclimated to a 1 × 10^7^ cells/mL concentration. Cells were then centrifuged for 5 min at 1200 rpm. Then, the supernatant was discarded, and the cell precipitate was kept. Each flow cytometry tube was labelled, and the groups were established as follows: control group, LPS-treated group, blank tube group, F4/80 single-stained tube, and CD86 single-stained tube. The appropriate F4/80 antibody (123115, BioLegend, San Diego, CA, USA) and CD86 antibodies (553692, BD Biosciences Pharmingen, San Jose, CA, USA) were added to each tube according to the experimental design. After adding the antibodies, the cells were left in the dark at 4 °C for 30 min. Thereafter, the cells were centrifuged one more time for 5 min at 1200 rpm. The supernatant was discarded. The cell precipitate was washed twice with 2 mL of PBS. A single-cell suspension was achieved by resuspending the cells in 200 µL of PBS for flow cytometry. All samples were analysed using FlowJo flow cytometry data analysis software, and the results were processed accordingly.

### Statistical Analysis

Statistical analyses were performed using GraphPad Prism 8 (GraphPad Software, San Diego, CA, USA). Data are presented as mean ± SD. Normality and homoscedasticity were assessed using the Shapiro-Wilk and Levene’s tests, respectively. Unpaired two-tailed Student’s t-test was used for two-group comparisons, with Welch’s correction applied when variances were unequal (Levene’s test, *P* < 0.05). One-way ANOVA followed by Tukey’s HSD post hoc test (for all pairwise comparisons, controlling familywise error rate) was used for multi-group comparisons when the overall effect was significant (*P* < 0.05). Exact *P* values and corresponding test statistics (t, df for t-tests; F, df for ANOVA) are provided in Supplementary Table S3. A two-tailed *P* < 0.05 was considered statistically significant.

## Results

### miR-155-5p is Upregulated in HF Hearts and Specifically Enriched in Infiltrating Macrophages

**Fig. 1:**
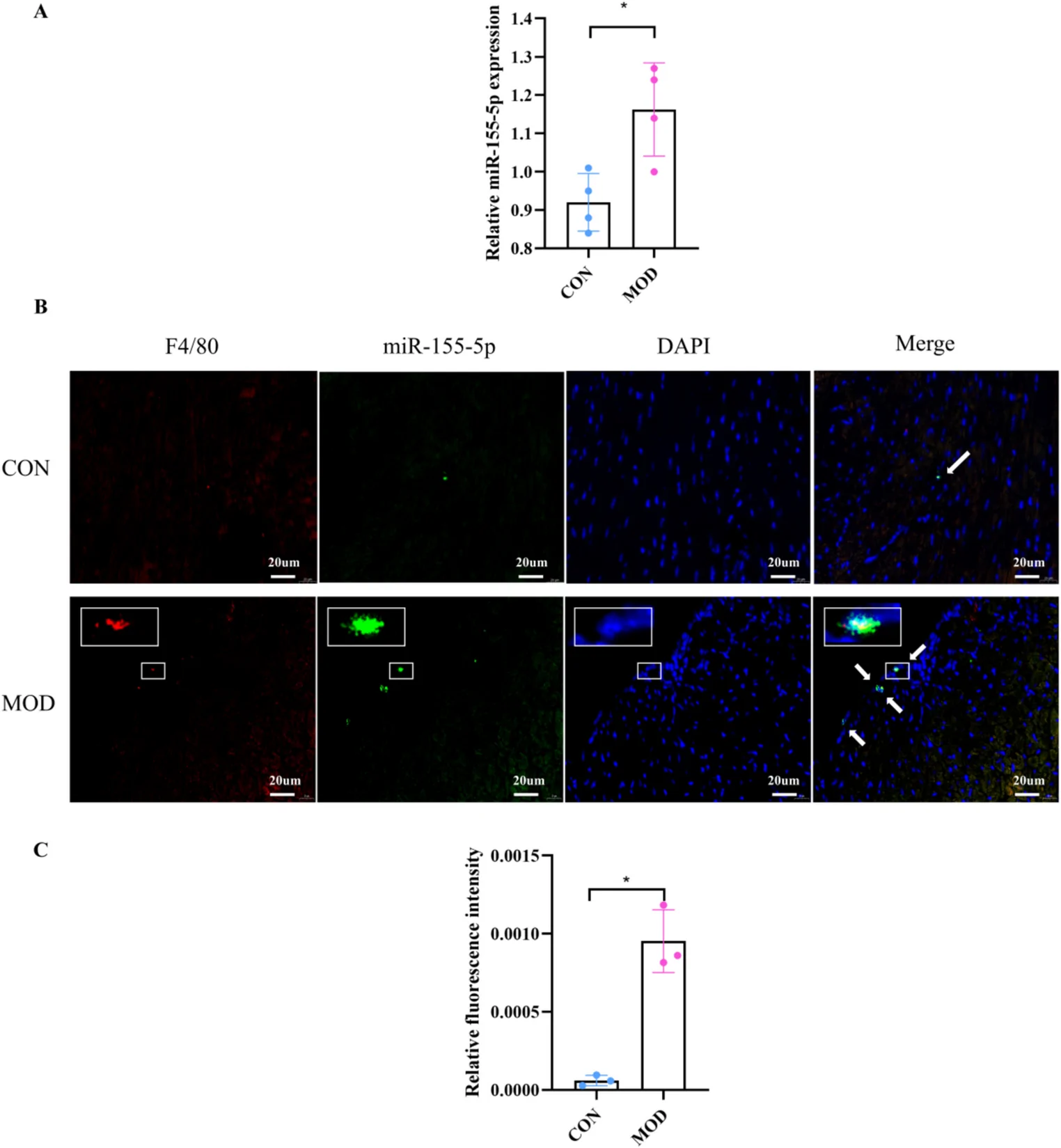
Elevated miR-155-5p levels in myocardial tissue of HF mice are primarily localized in macrophages. **A**: Relative expression levels of miR-155-5p in myocardial tissue quantified by RT-qPCR (*n* = 4); **B**: Representative immunofluorescence images of FISH/IF staining in the myocardium (×400). Macrophages stained with F4/80 antibody appeared red, while miR-155-5p stained green using a specific probe, and the nucleus was stained blue with DAPI. The white arrow identifies double-positive cells, which appear yellow. The representative cell is outlined by the white box; **C**: Quantification of relative fluorescence intensities between the CON and MOD group (*n* = 3). Notes: Data are presented as mean ± SD. (A) Unpaired two-tailed Student’s t-test; (C) Welch’s t-test. ^*^P < 0.05, relative to the CON group

miR-155-5p levels were significantly elevated in the hearts of mice in the MOD group compared with the CON group (Fig. 1A). Previous research has demonstrated ubiquitous miR-155-5p levels in adult mouse tissues; the highest production and distribution were observed in infiltrating macrophages [36–38]. Our previous work has shown that the macrophage phenotype in myocardial tissues of ISO-induced HF mice is predominantly M1 [30]. FISH/IF double-staining was used to localise miR-155-5p in myocardial tissues. Weak miR-155-5p signals were detectable in the CON group, primarily in F4/80^+^ macrophages. In the MOD group, miR-155-5p fluorescence intensity was significantly increased and remained co-localized with F4/80^+^ infiltrating macrophages (Fig. 1B). Quantitative analysis confirmed significantly higher miR-155-5p fluorescence intensity in the MOD group (Fig. 1C).

### SFI Improves Cardiac Function, Alleviates Myocardial Fibrosis, and Restores miR-155-5p/SOCS1 in HF Mice

**Fig. 2:**
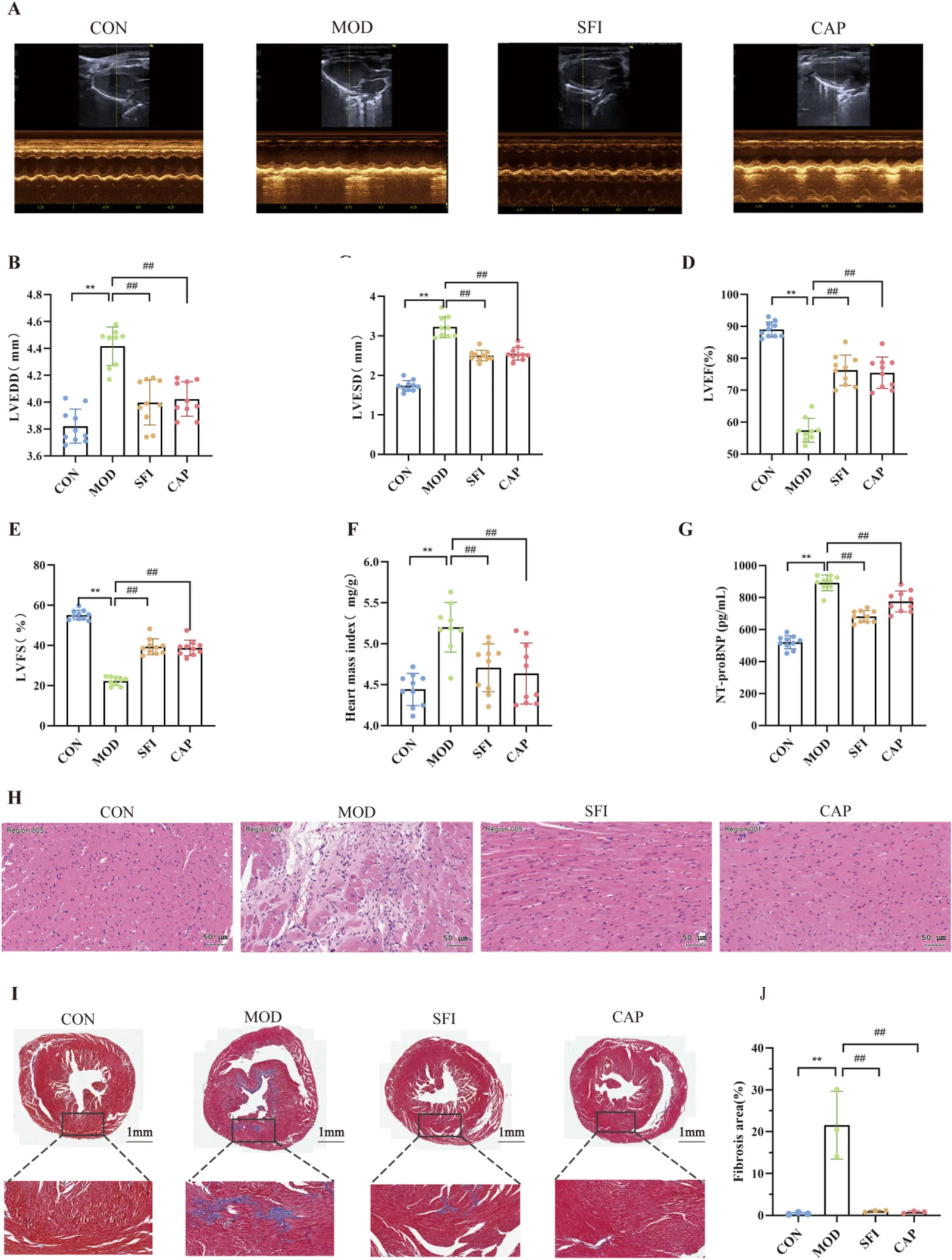
The inhibitory effect of SFI on myocardial fibrosis and its augmentation of cardiac performance. **A**: Schematic representation of echocardiography for each group; **B-E**: Echocardiographic parameters (except for the model group with *n* = 9, all the groups comprised *n* = 10); **F**: Heart mass index in each group (except for the model group with *n* = 9, all other groups had *n* = 10); **G**: Serum level of NT-proBNP quantified by ELISA (except for the model group with *n* = 9, all the groups comprised *n* = 10); **H**: Representative HE staining images of myocardial tissue (×200); **I**: The fibrosis representative images of heart sections using Masson’s staining (×200); **J**: Myocardial fibrosis rate (*n* = 3). Notes: Data were represented as mean ± SD. One-way ANOVA followed by Tukey’s HSD post hoc test. ^**^P < 0.01, relative to the CON group; ^##^*P* < 0.01, relative to the MOD group

To evaluate the therapeutic effects of SFI, ISO-induced HF mice were treated with SFI or CAP for 15 days. Representative echocardiographic images are illustrated in Fig. 2A. Compared with the CON group, the MOD group exhibited significantly increased LVEDD and LVESD, and decreased LVEF and LVFS. Both SFI and CAP significantly reversed these changes (Fig. 2B-E). The heart mass index and serum NT-proBNP levels were significantly elevated in the MOD group and reduced by SFI and CAP (Fig. 2F, G). HE and Masson staining showed extensive inflammatory cell infiltration and collagen deposition in the MOD group, which were markedly attenuated by SFI and CAP (Fig. 2H-J). These results indicate that SFI treatment significantly improved cardiac function in HF mice.

**Fig. 3:**
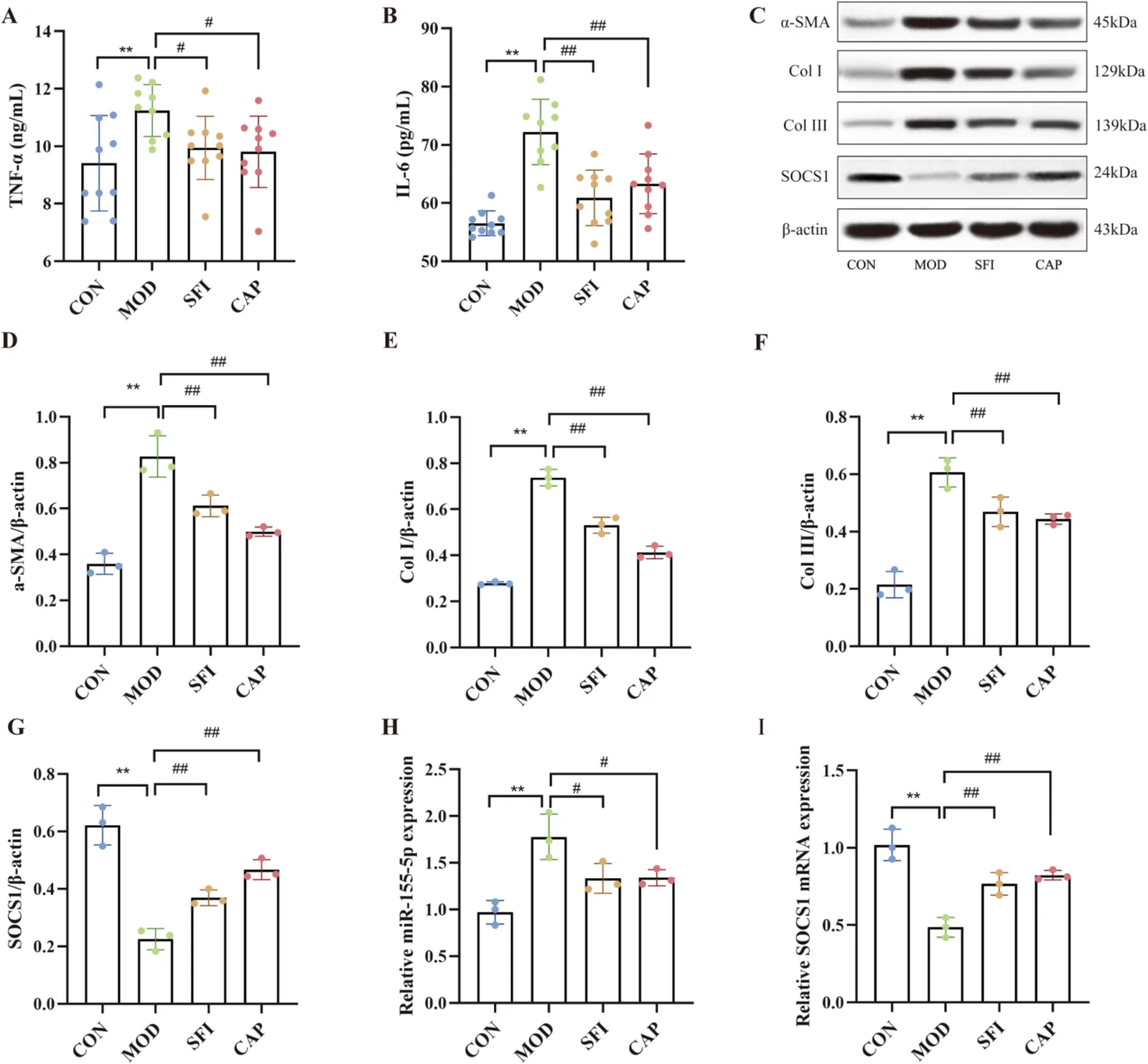
SFI attenuates inflammatory responses by inhibiting miR-155-5p levels and targets SOCS1 to ameliorate myocardial fibrosis. **A-B**: Serum level of TNF-α and IL-6, as quantified through ELISA (except for the model group with *n* = 9, all the groups comprised *n* = 10); **C**: Protein immunoblotting images of α-SMA, Col I, Col III, and SOCS1 in myocardial tissue; **D-G**: Comparing α-SMA, Col I, Col III, and SOCS1 protein content in myocardial tissue (*n* = 3); **H-I**: Relative levels of miR-155-5p, SOCS1, quantified through RT-qPCR (*n* = 3). Notes: Data were represented as mean ± SD. One-way ANOVA followed by Tukey’s HSD post hoc test. ^**^P < 0.01, relative to the CON group; ^#^*P* < 0.05, ^##^*P* < 0.01, relative to the MOD group

Earlier research has shown that miR-155-5p upregulates the production of TNF-α and IL-1β by downregulating the SOCS1 expression [39]. In this research, Serum TNF-α and IL-6 levels were significantly elevated in the MOD group compared with the CON group. Both SFI and CAP significantly reduced TNF-α (Fig. 3A) and IL-6 (Fig. 3B) levels. Western blot analysis revealed that α-SMA, Col I, and Col III levels were elevated in the MOD group, while SOCS1 expression was decreased. SFI and CAP treatments significantly suppressed α-SMA, Col I, and Col III protein levels and restored SOCS1 levels (Fig. 3C-G). RT-qPCR analysis showed that miR-155-5p levels were upregulated, whereas the SOCS1 mRNA levels were downregulated in the MOD group; both SFI and CAP interventions reversed these changes (Fig. 3H, I). These results demonstrate that SFI alleviates myocardial fibrosis and restores the miR-155-5p/SOCS1 axis in HF mice.

### SFI Suppresses the Levels of Exosome-derived miR-155-5p in M1 Macrophages

**Fig. 4:**
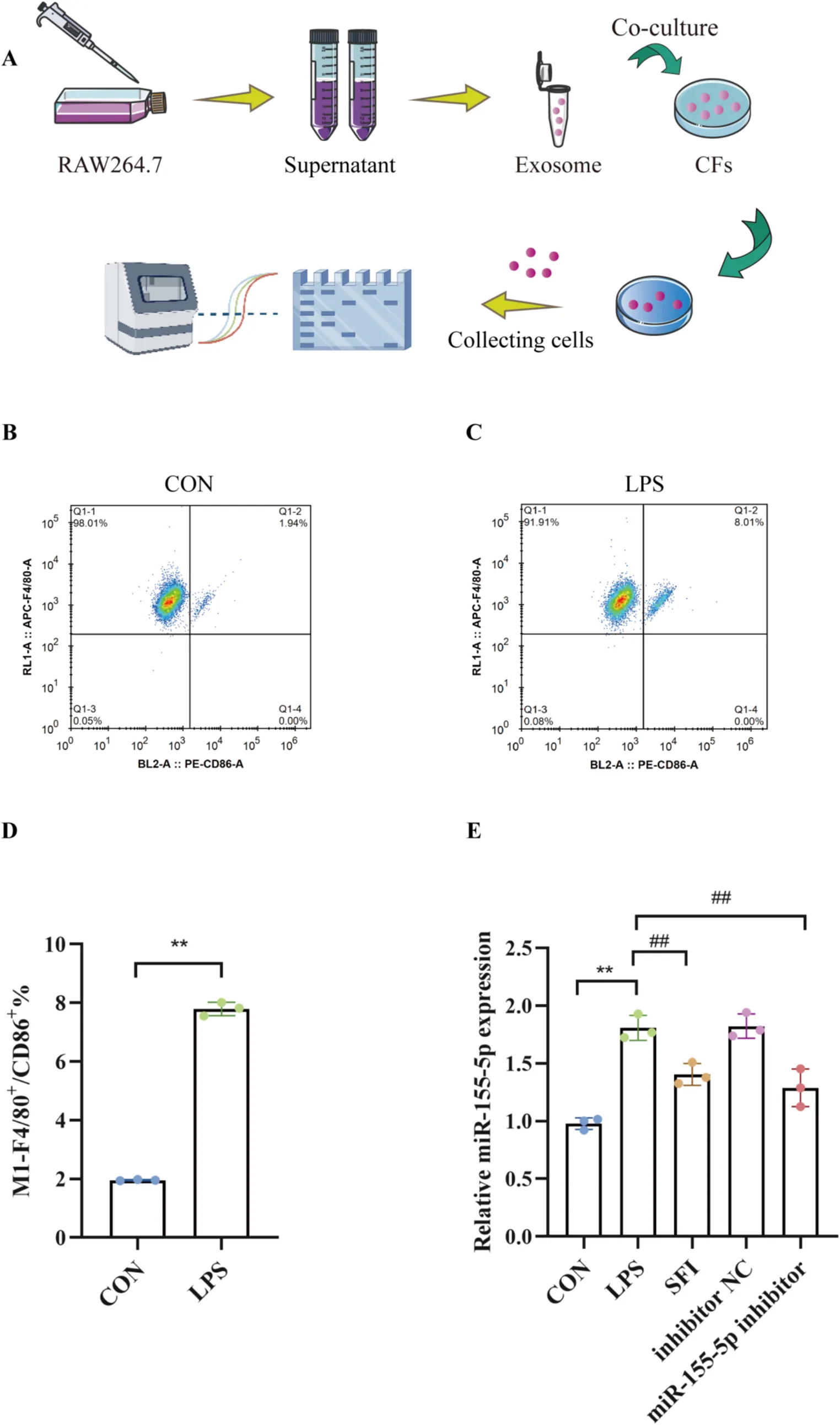
SFI treatment decreased miR-155-5p levels in exosomes derived from M1 macrophages. **A**: Flowchart of Cell Experiments; **B-D**: Proportions of F4/80 + CD86+ macrophages in CON and LPS groups were identified using flow cytometry (*n* = 3); **E**: Influence of SFI on miR-155-5p levels in exosomes from M1 macrophages (*n* = 3). Notes: The data were represented as mean ± SD. Comparisons between two groups were performed using unpaired two-tailed Student’s t-test. Multi-group comparisons were performed using One-way ANOVA followed by Tukey’s HSD post hoc test. ^**^P < 0.01, relative to the CON group; ^##^*P* < 0.01, relative to the LPS group

Given that SFI downregulated macrophage-associated miR-155-5p expression *in vivo*, we next asked whether SFI could directly regulate miR-155-5p expression in macrophage-derived exosomes. To this end, RAW264.7 macrophages were stimulated with LPS to induce M1 polarization, as outlined in Fig. 4A. Successful M1 polarization was confirmed by flow cytometry (Fig. 4B, D), and a non-cytotoxic SFI concentration of 4 µL/mL was selected by CCK-8 assay (Supplementary Fig. S5). Exosomes were then isolated from CON, LPS, SFI, miR-155-5p inhibitor NC, and miR-155-5p inhibitor groups. Exosome characterization confirmed successful isolation and that SFI did not alter exosome characteristics (Supplementary Fig. S6). RT-qPCR analysis revealed that exosomal miR-155-5p was significantly elevated in the LPS group compared with the CON group. Importantly, both SFI treatment and miR-155-5p inhibitor transfection markedly reduced exosomal miR-155-5p levels (Fig. 4E), demonstrating that SFI effectively suppresses miR-155-5p expression in M1 macrophage-derived exosomes.

### miR-155-5p Directly Targets SOCS1 to Promote CFs Activation

**Fig. 5:**
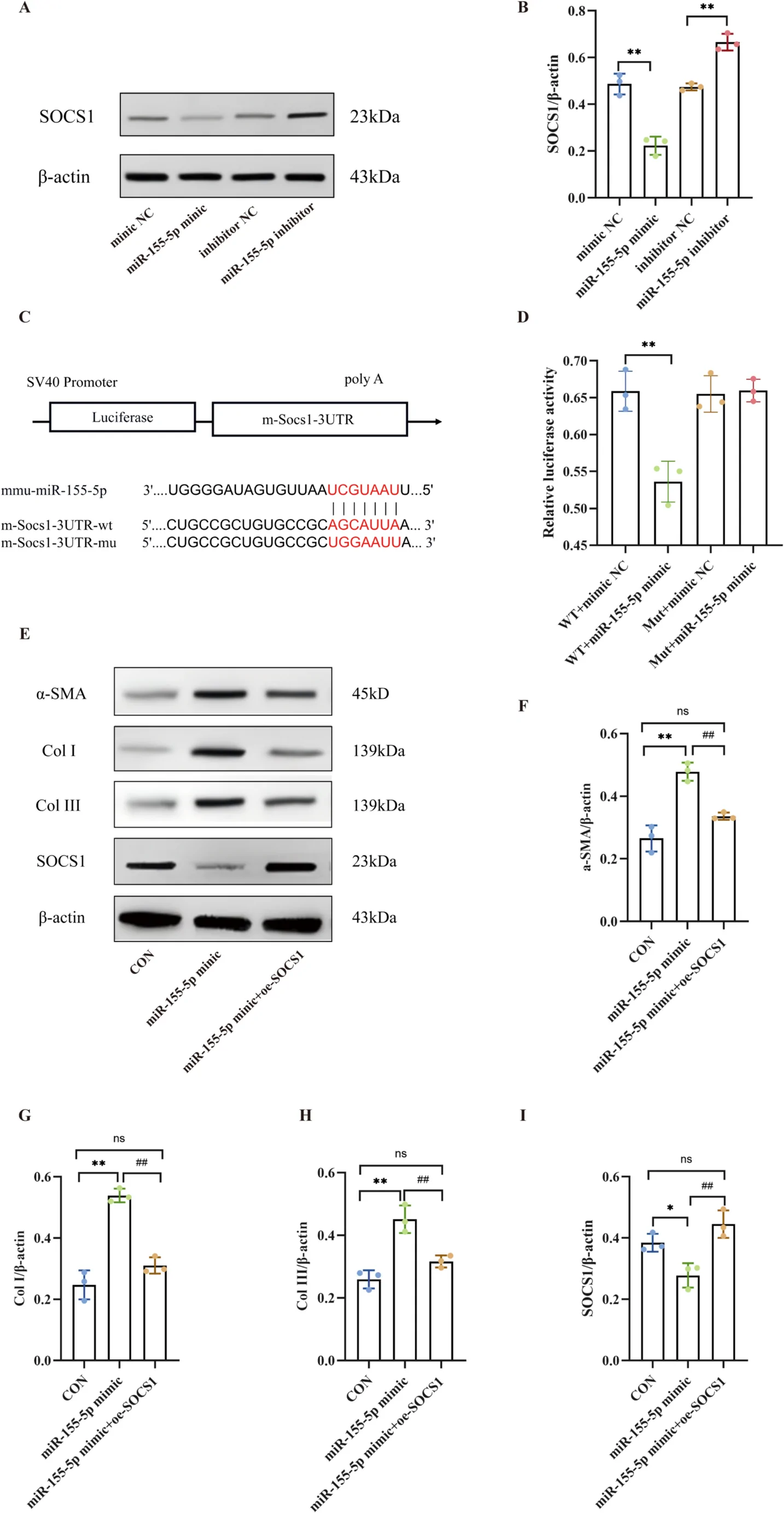
miR-155-5p directly targets SOCS1 to promote CFs activation. **A**,** B**: Western blot analysis of SOCS1 protein levels in CFs transfected with miR-155-5p mimic or inhibitor. β-actin was used as an internal control; **C**: Schematic representation of the dual-luciferase reporter constructs containing the WT or Mut Socs1 3′UTR; **D**: Dual-luciferase reporter assay in CFs co-transfected with miR-155-5p mimic and WT or Mut Socs1 3′UTR reporter plasmids; **E-I**: Rescue experiment. CFs were co-transfected with miR-155-5p mimic and/or oe-SOCS1 as indicated. **E**: Representative Western blots of α-SMA, Col I, Col III, and SOCS1; **F-I**: Quantification of α-SMA (F), Col I (G), Col III (H), and SOCS1 (I) protein levels. Notes: Data are presented as mean ± SD (*n* = 3). One-way ANOVA followed by Tukey’s HSD post hoc test. ^*^P < 0.05, ^**^P < 0.01, relative to the respective negative control (mimic-NC or inhibitor-NC in B; WT + mimic NC in D; CON in E-I). ^##^*P* < 0.01, relative to the miR-155-5p mimic group (E-I); ns, not significant relative to the the CON group

To determine whether miR-155-5p regulates SOCS1 expression in CFs, we transfected CFs with miR-155-5p mimic or inhibitor. miR-155-5p mimic significantly reduced SOCS1 protein levels compared with mimic NC, whereas miR-155-5p inhibitor significantly increased SOCS1 expression compared with inhibitor NC (Fig. 5A, B). A dual-luciferase reporter assay confirmed that miR-155-5p directly binds to the SOCS1 3′UTR (Fig. 5C), as miR-155-5p mimic significantly suppressed luciferase activity of the wild-type reporter, and mutation of the predicted binding site abolished this effect (Fig. 5D).

To confirm that miR-155-5p promotes CFs activation through targeting SOCS1, we performed a rescue experiment by co-transfecting CFs with miR-155-5p mimic and a SOCS1 overexpression plasmid lacking the 3′UTR (oe-SOCS1). Cells co-transfected with oe-NC and mimic-NC served as the CON group. Compared with the CON group, miR-155-5p mimic alone significantly downregulated SOCS1 and upregulated α-SMA, Col I, and Col III. Co-transfection of oe-SOCS1 significantly restored SOCS1 protein levels and reversed the miR-155-5p mimic-induced upregulation of α-SMA, Col I, and Col III to levels comparable to the CON group (Fig. 5E-I). Collectively, these results demonstrate that miR-155-5p directly targets SOCS1 to promote CFs activation.

### M1 Macrophage-derived Exosomes Transfer miR-155-5p to CFs and Induce CFs Activation by Targeting SOCS1

**Fig. 6:**
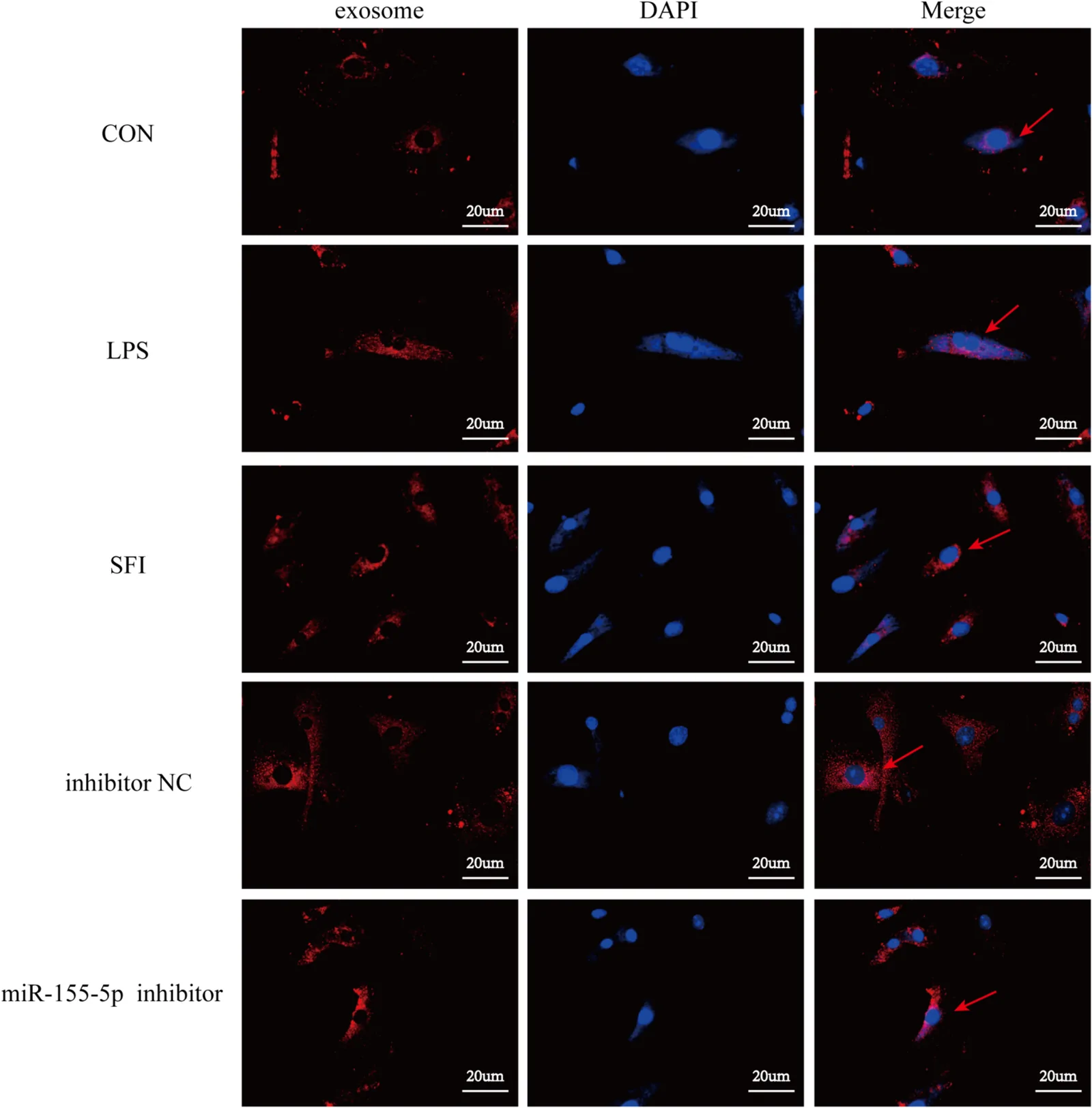
Exosomes derived from differently treated macrophages are internalised by CFs. Uptake of RAW264.7 cells-derived exosomes by CFs was visualised under fluorescence microscopy. PKH26-labeled exosomes appeared in red, while nuclear staining appeared in blue, with a magnification of ×400. Arrows indicate the distribution of red fluorescence from PKH26-labeled exosomes within the cytoplasm of CFs

To investigate the functional transfer of exosomal miR-155-5p, CFs were co-cultured with PKH26-labeled exosomes derived from macrophages under different treatments. Internalization of these exosomes was observed in CFs (Fig. 6). Subsequent co-cultures were conducted using 50 µg/mL exosomes for 24 h (Supplementary Fig. S7).

**Fig. 7:**
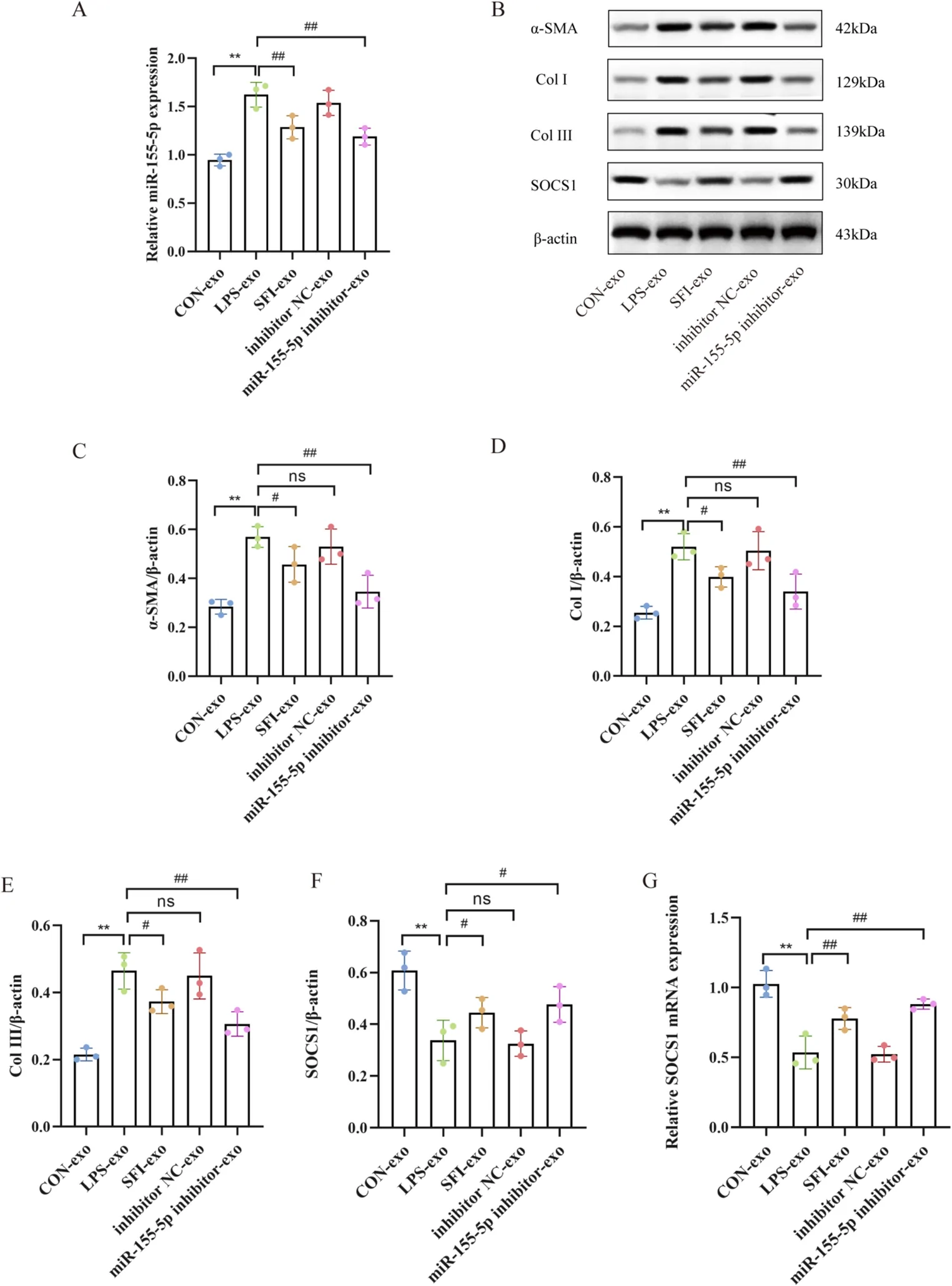
SFI inhibits the activation of CFs by targeting SOCS1 through the inhibition macrophage exosomal miR-155-5p. **A**: Influence of macrophage-derived exosomes on the levels of miR-155-5p in CFs; **B**: Protein immunoblotting images of α-SMA, Col I, Col III, and SOCS1 in CFs; **C-F**: Comparative analysis of the levels of α-SMA, Col I, Col III, and SOCS1 proteins in CFs across different groups; **G**: Comparative assessment of SOCS1 mRNA levels in CFs among various groups. Notes: The data were represented in the form of mean ± SD (*n* = 3). One-way ANOVA followed by Tukey’s HSD post hoc test. ^**^P < 0.01, relative to the CON-exo group; ^#^*P* < 0.05, ^##^*P* < 0.01, relative to the LPS-exo group

To determine whether exosomal miR-155-5p could be functionally transferred to CFs, we measured miR-155-5p levels in CFs after co-culture. Compared with the CON-exo group, miR-155-5p levels were significantly upregulated in CFs co-cultured with LPS-exo, whereas this upregulation was reversed by SFI-exo or miR-155-5p inhibitor-exo (Fig. 7A). To determine the functional consequences of exosomal miR-155-5p transfer, we assessed the levels of fibrosis-related proteins and SOCS1. Western blot analysis revealed that LPS-exo significantly upregulated the protein levels of α-SMA, Col I, and Col III, while downregulating SOCS1 protein in CFs (Fig. 7B-F). Consistent with this, SOCS1 mRNA levels were also decreased by LPS-exo (Fig. 7G). Treatment with SFI-exo or miR-155-5p inhibitor-exo restored SOCS1 expression and suppressed the upregulation of fibrotic markers (Fig. 7B-G). Together, these findings indicate that exosomes derived from M1 macrophages deliver miR-155-5p to CFs. This miRNA inhibits SOCS1 expression and promotes CFs activation, whereas SFI blocks this cascade by reducing exosomal miR-155-5p.

## Discussion

**Fig. 8:**
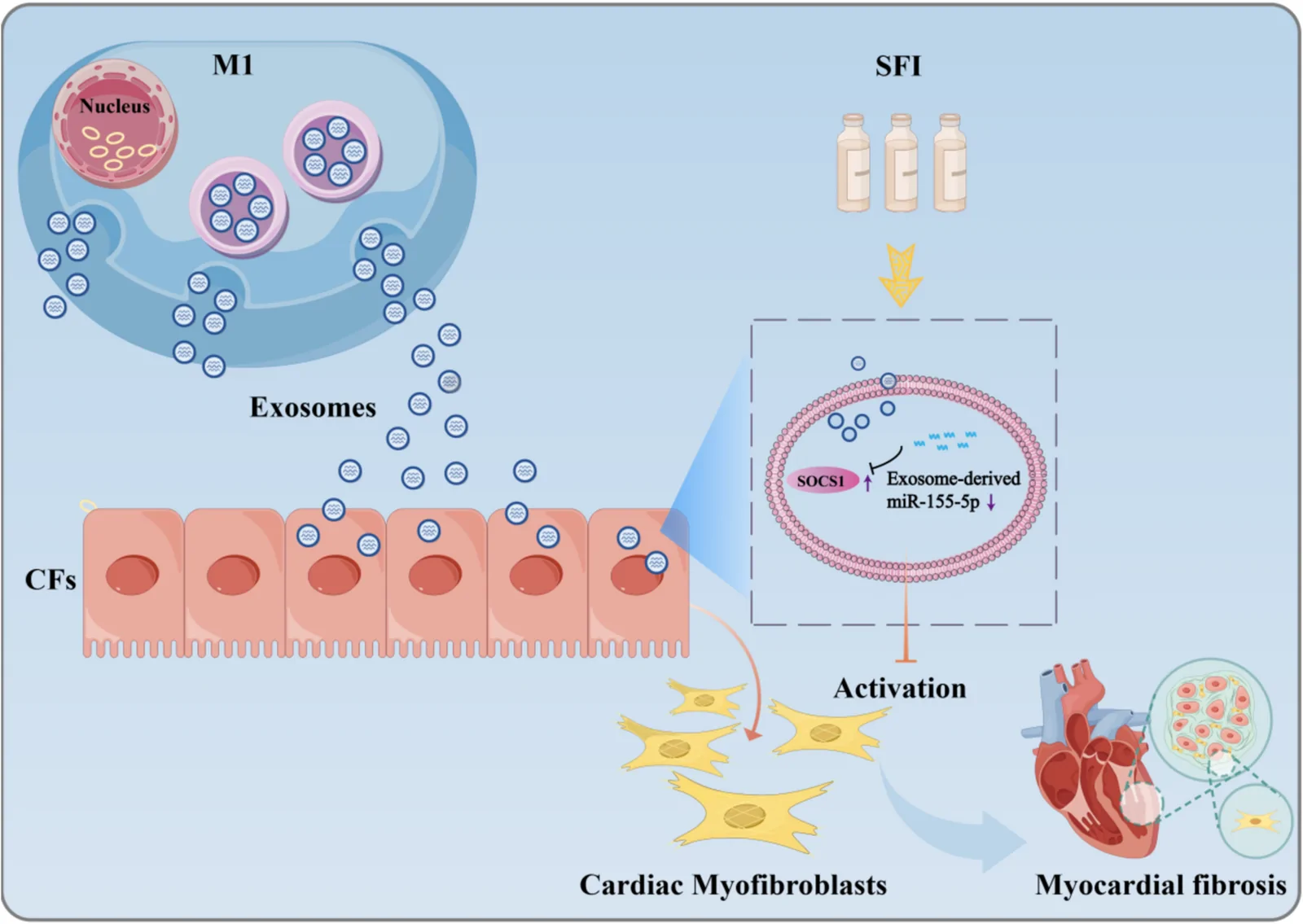
Summary of the role of SFI in inhibiting the activation of CFs and ameliorating myocardial fibrosis by downregulating miR-155-5p in M1 macrophage-derived exosomes. In the HF field, activated M1 macrophages release exosomes that are enriched with miR-155-5p and are subsequently transported to CFs, thereby facilitating their activation. SFI exerts its effects by suppressing miR-155-5p expression in these macrophage-derived exosomes, specifically targeting SOCS1 in CFs. This process leads to decreased activation of CFs, inhibition of myocardial fibrosis, and enhancement of cardiac function (This figure was drawn using Figdraw)

Myocardial fibrosis in HF represents a formidable medical challenge, as current therapeutic strategies remain limited. In this research, we utilized an ISO-induced mouse model of HF to elucidate a robust association between inflammation and myocardial fibrosis. Notably, we discovered a significant upregulation of miR-155-5p levels in the hearts of HF mice, primarily localised in the infiltrating macrophages. This aligns with the outcomes of previous studies that reported high miR-155-5p levels in myocardial tissues after acute infarction, particularly in pro-inflammatory macrophages activated by heart disease [40, 41]. Further investigation revealed that M1 macrophage-derived exosomes facilitated the activation of CFs via effectively delivering the miR-155-5p to upregulate SOCS1 levels. Administration of SFI markedly decreased miR-155-5p levels in both myocardial tissue and macrophage-derived exosomes, thereby inhibiting inflammatory responses, attenuating myocardial fibrosis, and enhancing cardiac function. These findings provide novel insights into the pathogenesis of HF and present potential therapeutic strategies (Fig. 8).

The clinical trials and animal studies have demonstrated the efficacy of SFI in improving cardiac function [42] and attenuating inflammatory responses in HF [43]. Consistent with these findings, our study in HF mouse models demonstrated that SFI significantly improved cardiac function, as indicated by decreased LVEDD, LVESD, and NT-proBNP levels, along with increased LVEF and LVFS. Additionally, SFI reduced the cardiac mass index and ameliorated myocardial histopathology. At the molecular level, SFI downregulated cardiac miR-155-5p expression and suppressed the upregulation of fibrosis markers (α-SMA, Col I, and Col III), indicating its role in attenuation of myocardial fibrosis. Myocardial fibrosis, driven largely by activated CFs, is a central pathological process in HF. Pro-inflammatory M1 Macrophages regulate myocardial fibrosis, in part, through the secretion of extracellular vesicles that mediate intercellular communication in the heart [44]. In particular, macrophage-derived exosomal miR-155-5p has been shown to promote cardiomyocyte pyroptosis and enhance inflammatory responses [45]. Furthermore, exosomes enriched with miR-155-5p from pro-inflammatory M1 macrophages exacerbate myocardial damage and cardiac dysfunction by impairing angiogenesis [46]. Our research confirms that macrophage exosomes transfer miR-155-5p to CFs, thereby stimulating their activation and promoting collagen synthesis. While other natural compounds, such as quercetin and crocetin, have been shown to attenuate cardiac fibrosis through distinct mechanisms—including the SIRT3/TGF-β pathway [47] and autophagy [48]—our findings reveal a novel mechanism by which SFI directly targets M1 macrophage-derived exosomal miR-155-5p. This is the first demonstration that SFI can reduce miR-155-5p levels in M1 macrophage exosomes and thereby inhibit CFs activation.

SOCS1 is a validated target of miR-155-5p [49], and its downregulation by miR-155-5p enhances the production of pro-inflammatory cytokines such as TNF-α and IL-1β [39]. As a key negative regulator of inflammatory signalling, SOCS1 directly inhibits JAK kinase activity to suppress IFN-γ-driven inflammatory cascades [50]. Recently, miR-155-5p/SOCS1 has emerged as a pro-fibrotic axis in the regulation of various diseases linked to fibrosis [19, 49], but its role in myocardial fibrosis remains less clear. Our findings demonstrate that M1 macrophage-derived exosomes deliver miR-155-5p to CFs, which directly targets SOCS1 and promotes CFs activation. This mechanism was further validated by dual-luciferase reporter and rescue experiments. The therapeutic effects of traditional Chinese medicine are frequently linked to their anti-inflammatory properties. In animal models, SFI treatment reduced cardiac levels of miR-155-5p while increasing SOCS1 expression, accompanied by decreased inflammatory cytokines (TNF-α, IL-6) and fibrosis markers (α-SMA, Col I, Col III). *In vitro*, exosomes from SFI-treated macrophages contained lower levels of miR-155-5p than those from LPS-stimulated macrophages. This reduction targeted and upregulated the SOCS1 pathway, inhibiting CFs activation in the inflammatory microenvironment. Collectively, our results indicate that the anti-inflammatory and antifibrotic impacts of SFI are partially mediated through inhibiting macrophage exosomal miR-155-5p expression and the targeting of the SOCS1 pathway to attenuate CFs activation.

We focused solely on miR-155-5p and its downstream target, SOCS1. However, this study did not explore some well-established fibrotic signalling pathways, particularly those involving the TGF-β/Smad pathway. It remains unclear whether the miR-155-5p/SOCS1 pathway directly mediates the activation of CFs or enhances the inflammatory cascade via other inflammatory pathways, including pyroptosis and cytosolic clearance. The absence of a normal control group treated with SFI alone is a limitation of this study, as it does not allow us to completely exclude potential direct effects of SFI on cardiac function or inflammatory status in healthy mice. We acknowledge this shortcoming and will include such a control group in future studies. Based on *in vitro* experiments, our research demonstrates the novel regulation of miR-155-5p in M1 macrophage exosomes by SFI. Additionally, it explores the preliminary insights into how SFI influences the interaction between macrophages and CFs via exosomal miR-155-5p. Future investigations are required to corroborate the performance of SFI in inhibiting CFs activation through macrophage exosomes in animal models via exosome reinfusion.

## Conclusion

In conclusion, this study identifies the miR-155-5p/SOCS1 axis as a direct regulatory mechanism of CFs activation. We demonstrate that M1 macrophage-derived exosomes transfer functional miR-155-5p to CFs, and that SFI inhibits this process by reducing exosomal miR-155-5p expression. The *in vivo* effects of SFI on myocardial fibrosis and SOCS1 restoration are consistent with this mechanism. These findings advance our understanding of exosome-mediated macrophage-fibroblast communication in myocardial fibrosis and provide a mechanistic basis for the therapeutic potential of SFI.

## Supplementary Information

Below is the link to the electronic supplementary material.

Supplementary Table S1 (DOCX 15.4 KB)

Supplementary Table S2 (DOCX 65.5 KB)

Supplementary Table S3 (XLS 37.5 KB)

Supplementary Fig. S1 (DOCX 1.12 MB)

Supplementary Fig. S2 (DOC 2.04 MB)

Supplementary Fig. S3 (DOC 24.1 MB)

Supplementary Fig. S4 (DOC 900 KB)

Supplementary Fig. S5 (DOC 1.12 MB)

Supplementary Fig. S6 (DOC 3.17 MB)

Supplementary Fig. S7 (DOC 1.40 MB)

## Data Availability

No datasets were generated or analysed during the current study.

## Abbreviations

α-SMA: α-smooth muscle actin
CHF: Chronic heart failure
CFs: Cardiac fibroblasts
Col I: Collagen I
Col III: Collagen III
CAP: Captopril
CON: Control
ELISA: Enzyme-linked immunosorbent assay
FISH: Fluorescence in situ Hybridization
IF: Indirect Immunofluorescence
IL-6: Interleukin-6
ISO: Isoprenaline
LPS: Lipopolysaccharide
LVEF: Left ventricular ejection fraction
LVFS: Left ventricular fractional shortening
LVEDD: Left ventricular end-diastolic diameter
LVESD: Left ventricular end-systolic diameter
MOD: Model
NT-proBNP: N-terminal pro-brain natriuretic peptide
RT-qPCR: Real-time quantitative PCR
SFI: Shenfu injection
SOCS1: Suppressor of Cytokine signalling 1
TNF-α: Tumor necrosis factor-α
UHPLC-QE-MS: Ultra-High Performance Liquid Chromatography coupled with Quadrupole-Orbitrap Mass Spectrometry
