# Supplementary material for: Shenfu Injection Inhibits Cardiac Fibroblast Activation and Improves Myocardial Fibrosis by Down-regulating M1 Macrophage-Derived Exosomal miR-155-5p
Source: Cardiovasc Drugs Ther. 2026 Jun 18;40(6):2117–34. doi: 10.1007/s10557-026-07901-7 (PMC13748244; doi:10.1007/s10557-026-07901-7)
Supplement: Supplementary file 1 [file 10557_2026_7901_MOESM1_ESM.docx]

Table S1

The information on raw botanical drugs in SFI

| Latin name | Plant parts | English name | Pinyin name | Voucher number | Place of origin | Proportion |
| --- | --- | --- | --- | --- | --- | --- |
| *Panax ginseng* C. A. Mey | Root | Red Ginseng Root | Hongshen | 13010560 | Origin of  place | 33.3% |
| *Aconitum carmichaelii* Debeaux | Tuber | Aconite  Tuber | Fuzi | 13010423 | Sichuan,  China | 66.6% |
