# Supplementary material for: Shenfu Injection Inhibits Cardiac Fibroblast Activation and Improves Myocardial Fibrosis by Down-regulating M1 Macrophage-Derived Exosomal miR-155-5p
Source: Cardiovasc Drugs Ther. 2026 Jun 18;40(6):2117–34. doi: 10.1007/s10557-026-07901-7 (PMC13748244; doi:10.1007/s10557-026-07901-7)
Supplement: Supplementary file 2 [file 10557_2026_7901_MOESM2_ESM.docx]

Table S2

The quality standard of SFI (batch number: 170902050).

| **metabolite** | **Chemical structure** | **Concentration** | **Standard regulatory concentration** |
| --- | --- | --- | --- |
| Ginsenoside Rg1 |  | 0.12 mg/mL | >0.04 mg/mL |
| Ginsenoside Re |  | 0.11 mg/mL | >0.02 mg/mL |
| Ginsenoside Rb1 |  | 1.0 mg/mL | 0.6–1.8 mg/mL |
| Benzoylmesaconine |  | 1.58 μg/mL | 0.50–4.50 μg/mL |
