# Supplementary material for: Shenfu Injection Inhibits Cardiac Fibroblast Activation and Improves Myocardial Fibrosis by Down-regulating M1 Macrophage-Derived Exosomal miR-155-5p
Source: Cardiovasc Drugs Ther. 2026 Jun 18;40(6):2117–34. doi: 10.1007/s10557-026-07901-7 (PMC13748244; doi:10.1007/s10557-026-07901-7)
Supplement: Supplementary file 4 [file 10557_2026_7901_MOESM10_ESM.doc]

**Fig. S7** Optimization of exosome intervention conditions on CFs. **A**: CFs were treated with macrophage-derived exosomes at different concentrations (0, 25, 50, and 100 μg/mL) for 24 h. Cell viability was assessed by CCK-8 assay. Cell viability was comparable between the CON group and the 25 or 50 μg/mL groups, but was significantly inhibited at 100 μg/mL. Therefore, 50 μg/mL was selected for subsequent experiments; B: CFs were treated with 50 μg/mL exosomes for 12, 24, or 48 h. Cell viability showed no significant difference at 12 h and 24 h compared with the time-matched CON group, but was significantly decreased at 48 h. Thus, 24 h was chosen as the optimal co-culture duration. Notes: Data were represented as mean ± SD (n=6). One-way ANOVA followed by Tukey's HSD post hoc test. ^**^*P* < 0.01, relative to the CON group; ns, not significant relative to the the CON group.
