# Supplementary material for: Shenfu Injection Inhibits Cardiac Fibroblast Activation and Improves Myocardial Fibrosis by Down-regulating M1 Macrophage-Derived Exosomal miR-155-5p
Source: Cardiovasc Drugs Ther. 2026 Jun 18;40(6):2117–34. doi: 10.1007/s10557-026-07901-7 (PMC13748244; doi:10.1007/s10557-026-07901-7)
Supplement: Supplementary file 5 [file 10557_2026_7901_MOESM5_ESM.doc]

Fig. S2 The UHPLC-QE-MS chromatogram illustration of three batches of SFI samples detected in positive ion mode (A-C) and negative ion mode (D-F).
