# Supplementary material for: Shenfu Injection Inhibits Cardiac Fibroblast Activation and Improves Myocardial Fibrosis by Down-regulating M1 Macrophage-Derived Exosomal miR-155-5p
Source: Cardiovasc Drugs Ther. 2026 Jun 18;40(6):2117–34. doi: 10.1007/s10557-026-07901-7 (PMC13748244; doi:10.1007/s10557-026-07901-7)
Supplement: Supplementary file 6 [file 10557_2026_7901_MOESM6_ESM.doc]

**Fig. S3** Iso-induced cardiac function decline in mice. **A**: Representative echocardiographic images of the CON and MOD groups; **B-E**: Quantitative analysis of echocardiographic parameters: LVEDD (B), LVESD (C), LVEF (D), and LVFS (E) (n=10). F: Serum NT-proBNP levels quantified by ELISA (n=10); **G**: Representative HE staining images of myocardial tissue (×200). Black arrow indicates cardiac oedema; Blue arrow indicates immune cell aggregation; **H**: Representative Masson's trichrome staining images of heart sections (×200). Blue indicates collagen deposition (fibrosis). Notes: The data were represented as mean ± SD. Unpaired two-tailed Student’s t-test; ^**^*P* < 0.01, relative to the CON group.
