# Supplementary material for: Shenfu Injection Inhibits Cardiac Fibroblast Activation and Improves Myocardial Fibrosis by Down-regulating M1 Macrophage-Derived Exosomal miR-155-5p
Source: Cardiovasc Drugs Ther. 2026 Jun 18;40(6):2117–34. doi: 10.1007/s10557-026-07901-7 (PMC13748244; doi:10.1007/s10557-026-07901-7)
Supplement: Supplementary file 7 [file 10557_2026_7901_MOESM7_ESM.doc]

**Fig. S4** Validation of miR-155-5p transfection efficiency. **A**: RAW264.7 macrophages were transfected with miR-155-5p inhibitor or inhibitor NC. miR-155-5p levels were significantly decreased in the inhibitor group compared with the inhibitor NC group (n = 3); **B**: CFs were transfected with miR-155-5p mimic, miR-155-5p inhibitor, or their respective negative controls (mimic NC, inhibitor NC). miR-155-5p levels were significantly increased in the mimic group compared with the mimic NC group, and significantly decreased in the inhibitor group compared with the inhibitor NC group (n = 3). U6 was used as an internal control. Note: Data are presented as mean ± SD. One-way ANOVA followed by Tukey's HSD post hoc test. ^**^*P* < 0.01, relative to the respective negative control group (mimic NC, inhibitor NC); ns, not significant relative to the the CON group.
