# Supplementary material for: Shenfu Injection Inhibits Cardiac Fibroblast Activation and Improves Myocardial Fibrosis by Down-regulating M1 Macrophage-Derived Exosomal miR-155-5p
Source: Cardiovasc Drugs Ther. 2026 Jun 18;40(6):2117–34. doi: 10.1007/s10557-026-07901-7 (PMC13748244; doi:10.1007/s10557-026-07901-7)
Supplement: Supplementary file 8 [file 10557_2026_7901_MOESM8_ESM.doc]

**Fig. S5** Screening of the optimal concentration of SFI on M1 macrophages using the CCK-8 assay. RAW264.7 macrphages were treated with varying concentrations of SFI (0, 1, 2, 4, 8, 12, 16 μL/mL) for 24 h. Cell viability was determined by CCK-8 assay. The cell viability was markedly inhibited when SFI reached 8 µL/mL, so 4 µL/mL was chosen for subsequent studies. Notes: Data are presented as mean ± SD (n=5). One-way ANOVA followed by Tukey's HSD post hoc test. ^**^*P* < 0.01, compared with the LPS group (0 μL/mL).
