# Supplementary material for: Shenfu Injection Inhibits Cardiac Fibroblast Activation and Improves Myocardial Fibrosis by Down-regulating M1 Macrophage-Derived Exosomal miR-155-5p
Source: Cardiovasc Drugs Ther. 2026 Jun 18;40(6):2117–34. doi: 10.1007/s10557-026-07901-7 (PMC13748244; doi:10.1007/s10557-026-07901-7)
Supplement: Supplementary file 9 [file 10557_2026_7901_MOESM9_ESM.doc]

**Fig. S6** Characterization of exosomes derived from LPS- and SFI-treated RAW264.7 macrophages.**A**: Representative transmission electron microscopy (TEM) images of exosomes from the LPS and SFI groups, showing the typical cup-shaped morphology (indicated by black arrows). Scale bar: 200 nm; **B**: Particle size distribution measured by nanoparticle tracking analysis (NTA). The size distribution profiles were similar between the two groups, with a peak diameter of approximately 100–150 nm; **C**: Western blot analysis of exosomal markers (TSG101, ALIX, and CD63). These markers were detected in both groups, confirming that SFI treatment did not alter exosomal characteristics.
